# Potential Push-Pull Carbon Superbases Based on Methyl Substitution of Rare Tautomers of Imines

**DOI:** 10.3390/molecules30030474

**Published:** 2025-01-22

**Authors:** Ewa Daniela Raczyńska, Jean-François Gal, Pierre-Charles Maria

**Affiliations:** 1 Department of Chemistry, Warsaw University of Life Sciences (SGGW), ul. Nowoursynowska 159c, 02-776 Warsaw, Poland; 2 Institut de Chimie de Nice, UMR 7272, Université Côte d’Azur, Parc Valrose, 06108 Nice, France; jean-francois.gal@univ-cotedazur.fr (J.-F.G.); pierre-charles.maria@univ-cotedazur.fr (P.-C.M.)

**Keywords:** tautomeric superbases in vacuo, prototropic equilibria, alkylated derivatives, PA/GB estimations, G*n* and/or DFT studies

## Abstract

Push-pull imines with strong electron donor group(s) display exceptional basicity in the gas phase. Most of them do not exhibit prototropic tautomerism, and gas-phase acid-base equilibria have been already well described and reviewed. Some questions remain for tautomeric systems, particularly for their uncommon forms. As shown by quantum-chemical calculations, some often-neglected tautomers display higher basicity than the thermodynamically favored forms. However, their participation in tautomeric mixtures being in equilibrium is negligible, and their basicity can be impossible to measure in the gas phase by the equilibrium method. During this work, we examined the gas-phase proton basicity for some acyclic and cyclic push-pull organic bases containing the tautomeric amidine or guanidine group. By quantum-chemical calculations, we confirmed the existence of very low amounts of rare tautomeric forms, in particular, those bearing a methylidene (=CH_2_) group. We also demonstrated that the alkyl derivatives of rare tautomers, being free of prototropy, can be good candidates as very strong push-pull C bases, i.e., bases protonated on the =CH_2_ group.

## 1. Introduction

Push-pull organic Brønsted bases, such as nitriles, amidines, guanidines, biguanides, vinamidines, phosphazenes, guanidinophosphazenes, etc., display a very strong basicity in the gas phase [1,2,3,4]. They contain one or more electron-donating (D, pushing) group(s) {R_2_N, R_2_NC(R)=N, (R_2_N)_2_C=N, R_3_P=N, R_2_NP(R_2_)=N, (R_2_N)_2_P(R)=N and/or (R_2_N)_3_P=N, R: H, acyclic or cyclic alkyl} attached directly to an electron-withdrawing (A, pulling) group (C≡N or C=N with N as the protonation site) or separated by a π-electron cyclic or acyclic spacer, including aromatic ring(s), e.g., D–A, D–(π-cycle)_n_–A, D–(aryl)_n_–A, D–{C(R)=C(R)}_n_–A, D–{C(D)=C(R)}_n_–A, D–{C(D)=C(D)}_n_–A, D–{C(R)=N}_n_–A, D–{C(D)=N}_n_–A, D–{P(R_2_)=N}_n_–A, D–{(R)P(D)=N}_n_–A, and D–{P(D_2_)=N}_n_–A (n: 1, 2, 3, etc.). The superbasicity of these push–pull molecules is a consequence of very strong n-π linear-, Y-, or cross-conjugation (resonance effect) between the D and A groups. When a stronger pushing group is linked, the pulling site has a stronger basicity [1,2]. However, the increase in basicity by increasing the number of pushing groups reaches some limits when reaching the superbasicity range [3]. After protonation, the monocationic AH^+^ group (C≡NH^+^ or C=NH^+^), possessing enhanced electron-withdrawing properties, through protonation, usually increases π-electron delocalization when going from neutral to protonated form [1,4]. For more details on various types of conjugations between D and A groups; see Ref. [2].

**Figure 1:**
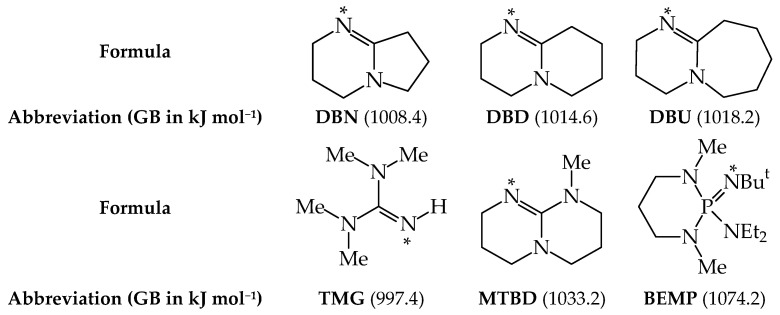
Examples of push-pull strong N bases (free of prototropy) and their experimental GBs (in kJ mol^−1^, taken from Ref. [2]). Site of protonation indicated by an asterisk (*).

The most documented examples of superbases used as catalysts in organic syntheses are as follows: amidines like **DBN** (1,5-diazabicyclo[4.3.0]non-5-ene), **DBD** (1,5-diazabicyclo[4.4.0]dec-5-ene), and **DBU** (1,8-diazabicyclo[5.4.0]undec-7-ene), guanidines like **TMG** (N^1^,N^1^,N^3^,N^3^-tetramethylguanidine) and **MTBD** (7-methyl-1,5,7-triazabicyclo[4.4.0]dec-7,9-ene), and the phosphazene **BEMP** (2-*tert*-butylimino-2-diethylamino-1,3-dimethylperhydro-1,3,2-diazaphosporine). Their structures and thermodynamic basicity data are given in Figure 1. For more examples of push-pull N superbases, see Refs [1,2,3,4]. The experimental gas-phase proton basicities (GBs, see the Section 3 for its definition), measured mainly by the equilibrium method and recently reviewed [2], are higher than that (GB 995.8 kJ mol^−1^) of the so-called proton sponge **DMAN** {1,8-bis(dimethylamino)naphthalene}.

Most push-pull superbases of known experimental GBs do not exhibit prototropic tautomerism (prototropy), and the site of monoprotonation reaction is well determined for them [1,2,3,4]. For amidines and guanidines, the pulling atom (imino N) is preferentially protonated. Its strong basicity in the gas phase (exceptionally high GBs) originates mainly from n-π linear- or Y-conjugation between amino and imino groups. These groups play the following roles in proton-transfer equilibria: electron-donor substituent(s) and electron-acceptor protonation function, respectively. The basicity of the amino N atom(s) in push-pull imino superbases, amidines, and guanidines, is lower than that of the monoprotonation site by more than 100 kJ mol^−1^ [2].

**Figure 2:**
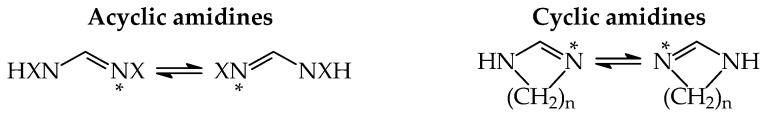
Two identical tautomers of symmetrically substituted push-pull tautomeric acyclic and cyclic amidines (* indicates protonation site).

For symmetrically substituted systems with the tautomeric amidine group –NH–CH=N– (B_1_) ⇌ –N=CH–NH– (B_2_), the tautomeric mixtures consist mainly of two identical tautomeric forms (50% + 50% = 100%) with the imino N atom as the site of monoprotonation. The monoprotonation of such a system (B_1_ ⇌ B_2_) always leads to one common monocation {–NHCHNH–}^+^ (BH^+^). In the reverse reaction, the monodeprotonation of BH^+^ leads to two tautomers, B_1_ and B_2_, with identical structures. Tautomeric conversion together with reversible protonation-deprotonation reactions gives the complete acid-base equilibria for symmetrically N,N’-disubstituted amidines. Some examples of simple acyclic and cyclic derivatives are given in Figure 2. They were only theoretically investigated. For example, the proton affinity (PA, see the Section 3 for its definition) of acyclic formamidine is lower than 1000 kJ mol^−1^ (949.4 [5] and 948.2 kJ mol^−1^ [6] at the G2 and G4MP2 levels, respectively) because of the absence of charge stabilization by alkyl groups (G2 and G4MP2 are abbreviations of various variants of Gaussian-*n* theory; see the list of abbreviations). However, its basicity is considerably stronger than that of methylimine, ammonia, and methylamine (PA: 852.9, 853.6, and 899.0 kJ mol^−1^, respectively [7]).

In the case of the cyclic amidines given in Figure 2, PAs vary as follows: 928 (*n* = 1), 973 (*n* = 2), and 1007 (*n* =3) kJ mol^−1^ at the G4MP2 level [6]. With an increase in the number (*n*) of the methylene groups in the ring from one to two, and then from two to three, the PA values increase by 45 and 34 kJ mol^−1^, respectively. This suggests that the PA increase for the imino N atom with ring size may be subject to leveling.

An analogous phenomenon of prototropy takes place for symmetrically substituted guanidines containing two amino groups with one H atom at each of them and substituent X not engaged in prototropy. For these guanidines, three identical tautomers are possible, XHN–C(NHX)=NX ⇌ XN=C(NHX)–NHX ⇌ XHN–C(=NX)–NHX, in which the imino N atom is the site of monoprotonation. For example, in the case of their parent compound, H_2_N–C(NH_2_)=NH ⇌ HN=C(NH_2_)–NH_2_ ⇌ H_2_N–C(=NH)–NH_2_, both experimental (PA 986.3 kJ mol^−1^ [7]) and quantum-chemical calculations (PA 986.2 [8] and 987.9 kJ mol^−1^ [6] at the G2 and G4MP2 levels, respectively) confirmed that n-π Y-conjugated guanidine is a stronger base than n-π linear-conjugated acyclic formamidine containing only one amino (pushing) group.

**Figure 3:**
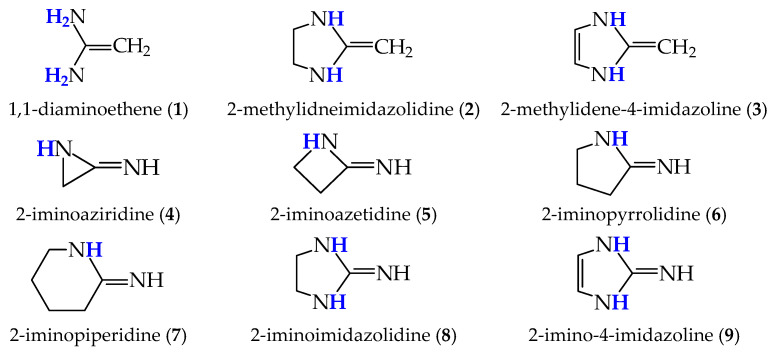
Selected rare tautomers of tautomeric strong bases. Tautomeric proton(s) indicated in blue bold.

A different situation occurs for unsymmetrically substituted tautomeric systems containing the amidine and/or guanidine group with at least one H at amino N. Their tautomeric mixtures can contain two or more tautomers displaying different basicity strengths and different contributions in the tautomeric mixtures. However, in the literature, we can find many reports focusing mainly on rare tautomers for which an exceptionally high basicity has been predicted by using quantum-chemical methods, see, for example, Refs [4,6,9,10,11,12,13,14,15,16,17,18]. Figure 3 shows some rare tautomers of simple acyclic and cyclic amidines (**1**–**7**) and cyclic guanidines (**8**,**9**). Only for compound **1**, acetamidine isomers have been examined in detail [9]. Prototropic tautomerism in cyclic push-pull imines (**2**–**9**) has never been taken into account in the theoretical reports of Maksić, Koppel, Bouchoux, and their co-workers [4,6,10,11,12,13,14,15,16]. In the same vein, Rouhani and co-workers [17,18] prolonged quantum-chemical calculations in the domain of superbasicity.

Acyclic structure **1** (Figure 3) is an enamino form of acetamidine, H_2_N–C(CH_3_)=NH ⇌ HN=C(CH_3_)–NH_2_. Note that Maksić and Vianello, in Ref. [10], considered 1,1-diaminoethene (**1**) and acetamidine as different compounds. On the other hand, Miranda et al. [19], investigating a series of diaminoethenes by using quantum-chemical methods, examined only **1**, ignoring its tautomeric conversion to acetamidine. Amidines **4**–**7** were studied by Bouchoux and Eckert-Maksić [6]. Although Bouchoux and his co-workers analyzed earlier isomerism and intramolecular proton transfers for amino acids [20], they also ignored prototropy for **4**–**7** [6] and investigated gas-phase proton basicity parameters for only one tautomer. Guanidine tautomers, in particular, the structures with five-membered rings (**8** and **9**), were frequently considered by Koppel, Leito, Maksić, Schröder, and their co-workers in propositions of new structures for strong N bases [11,12,13,14]. Acyclic (**1**) and cyclic derivatives of diaminoethenes (**2** and **3**) have been also included in the structures of superbases [4,10,12,15,16,17,18] without consideration of prototropy.

Considering the incomplete literature data for tautomeric push-pull strong organic bases, first, we analyzed the tautomeric mixtures containing all potential tautomers for derivatives **1**–**9**. We estimated the participation of individual isomers in the tautomeric mixtures, and their microscopic gas-phase proton basicities for the potential sites of protonation. We also examined their macroscopic PA/GB values, which can give some information on experimental PAs/GBs. Finally, we indicated the favored structures that consist mainly of tautomeric mixtures, which, in turn, dictate their PAs/GBs. Our investigations for tautomeric systems were carried out using quantum-chemical methods (G*n* and/or DFT; see the explanation in the list of abbreviations). In this way, we could quantitatively indicate that rare tautomers, very frequently considered in the literature as thermodynamically stable compounds and proposed as candidates for new superbases, have exceptionally high Gibbs energies in comparison to favored tautomers. These rare tautomers can be difficult or even impossible to generate for basicity measurements. In conditions of acid-base reactions in the gas phase, their trace amounts can be protonated to monocations. These ionic species in proton-transfer equilibria are mainly conjugated with tautomers of lower Gibbs energy.

Since rare tautomers exhibit, in general, higher basicity in the gas phase than favored tautomers, alkyl derivatives of rare tautomers could be the most prominent derivatives for the extension of the superbasicity scale. For this reason, in this work, we investigated some methyl derivatives of rare isomers that could be synthesized in the future with the aim of generating strong organic bases. In the following, the name “labile” refers to the protons that can be engaged in prototropy. To avoid prototropy in such a structure, all labile (tautomeric) protons were structurally replaced by Me group(s). Investigations on the structure and gas-phase proton basicity for these alkyl derivatives were carried out using a DFT approach. In the future, they may find many applications, e.g., in organic syntheses as organocatalysts, in the field of material science, for CO_2_ capture, and as ionic liquids, like strong push-pull N-bases (see in Ref. [1]).

## 2. Results and Discussion

### 2.1. Basicity of Acyclic Tautomeric System ***1*** Containing the Amidine Group

1,1-Diaminoethene (**1**) is a rare (experimentally undetectable) tautomer of acetamidine, H_2_N–C(CH_3_)=NH (B_1_) ⇌ HN=C(CH_3_)–NH_2_ (B_2_) ⇌ H_2_N–C(=CH_2_)–NH_2_ (B_3_), for which two types of prototropic equilibria take place: one amino-imine conversion in the amidine group (B_1_ ⇌ B_2_) and the other imino-enamine tautomerism in the methylimine part (B_1_ ⇌ B_3_ and B_2_ ⇌ B_3_). Consequently, acetamidine consists of three tautomeric forms: B_1_, B_2_, and B_3_ [9]. Two of them (B_1_ and B_2_) are structurally identical, while the third tautomer B_3_ is completely different than B_1_/B_2_. Additionally, configurational isomerism occurs about the double C=N bond in tautomers B_1_ and B_2_; thus, two extreme configurational isomers are possible (*E* and *Z*) [21].

For *E*- and *Z*-B_1_/B_2_ of **1**, the imino N atoms are the favored protonation sites, and the methylene C atom is preferentially protonated for B_3_ [9]. Monoprotonation of all isomers at the favored sites of protonation leads to one common monocation, H_2_NC(CH_3_)NH_2_^+^ (BH^+^). It should be mentioned here that protonation at the amino N atoms in B_1_/B_2_ and B_3_ lead to cations of considerably higher energies than that of BH^+^ (by ca. 120 and 150 kJ mol^−1^, respectively, at the G2MP2 level [9]). Hence, these cations can be neglected in the isomeric mixture of the monocationic form of acetamidine.

**Figure 4:**
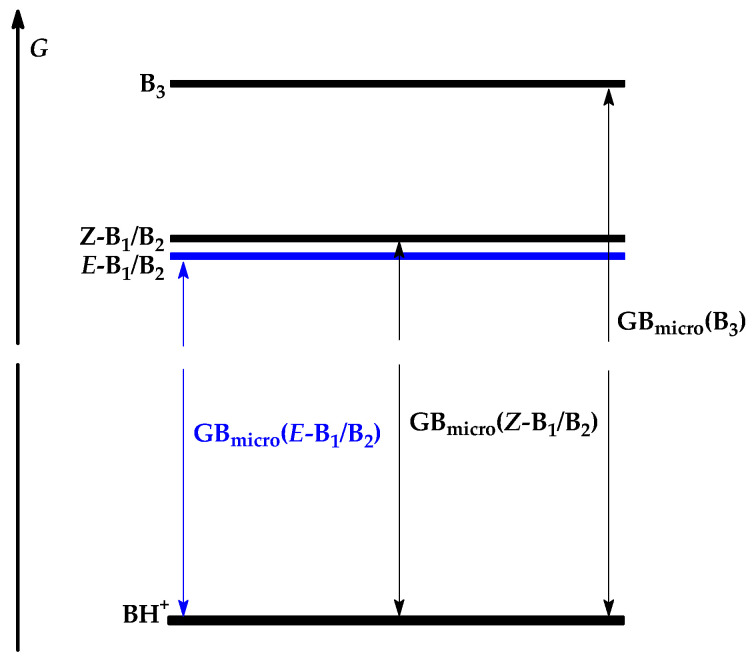
General scheme of the relation between Gibbs energies (*G*) for three isomers of acetamidine **1** (*E*-B_1_/B_2_, *Z*-B_1_/B_2_, and B_3_) and its protonated form (BH^+^) and between their microscopic gas-phase proton basicities (GB_micro_). On the *G* scale, the position of the favored isomer and of its GB_micro_ is indicated in blue.

Figure 4 illustrates the general scheme showing the relation between the Gibbs energies (*G*) calculated at the DFT or G*n* levels for the neutral isomers of acetamidine **1** (*E*-B_1_/B_2_, *Z*-B_1_/B_2_, and B_3_) and its monoprotonated form (BH^+^). The favored tautomer-rotamer *E*-B_1_/B_2_, having the lowest Gibbs energy, always possesses the lowest microscopic GB value, while the rare tautomer B_3_, displaying the highest Gibbs energy, has the highest microscopic GB value. An analogous picture occurs for the enthalpies (*H*) and microscopic PAs of *E*-B_1_/B_2_, *Z*-B_1_/B_2_, and B_3_. The absolute values of the relative entropy term for the tautomers B_1_/B_2_ and B_3_ are very small (*T*Δ*S* < 3 kJ mol^−1^ at 298.15 K). They are considerably smaller than their relative energies, enthalpies, and Gibbs energies (Δ*E*_0_, Δ*H*_298_, and Δ*G*_298_ ≥ 40 kJ mol^−1^). The relative entropy term for the configurational isomers *E* and *Z* of B_1_/B_2_ is close to zero.

The theoretical results calculated in this work for the three isomers of acetamidine **1** and their mixture at the DFT and G*n* levels, and compared to some literature data, are summarized in Table 1. The percentage contents estimated at different levels of theory for individual isomers show evidently that the *E*-isomer (84–86%) of two identical tautomers B_1_ and B_2_ predominates in the isomeric mixture of **1** in the gas phase. The amounts of the *Z*-isomer (14–16%) are considerably smaller, but both isomers consist of the tautomeric mixture of **1** in equilibrium. This trend is completely different in the solid state. The *Z*-isomer is favored for acetamidine being in the crystal form [21].

The percentage contents of B_3_ calculated at different levels of theory are exceptionally small (<5 × 10^−6^%), and this tautomer can be neglected in an equilibrium tautomeric mixture. The contribution of B_3_ to the macroscopic PA and GB values is not significant. Therefore, the calculated macroscopic basicity parameters are close to those of microscopic ones found for the favored *E*-isomer of B_1_ and B_2_, and also to the experimental PA and GB values [7,9]. They are very different from those of B_3_ due to exceptionally high values of Δ*H*_298_ and Δ*G*_298_. This means that in experiments on protonation/deprotonation equilibria, it will be difficult or even impossible to select B_3_ and measure its gas-phase proton basicity. Exceptionally small amounts of B_3_ after protonation leading to the common monocation BH^+^ will quickly turn into favored B_1_/B_2_ in the reverse deprotonation reaction.

### 2.2. Basicity of Cyclic Tautomeric Systems ***2***–***7*** Containing the Amidine Group

**Figure 5:**
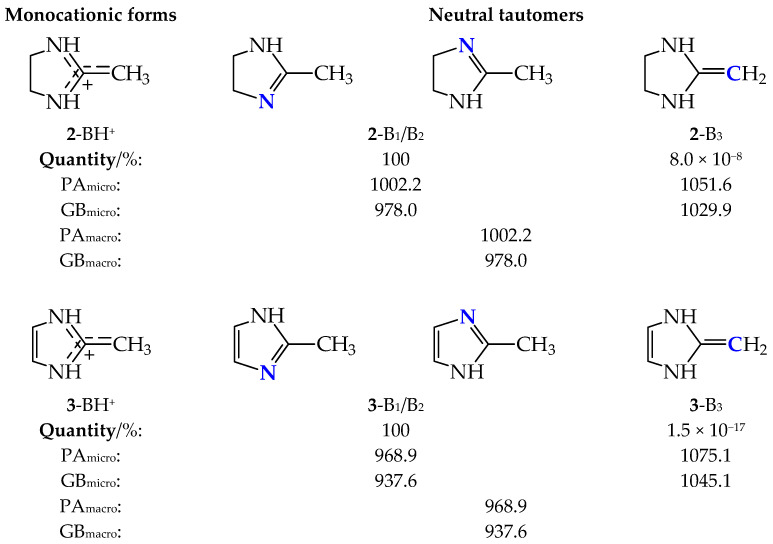
Structures of monocationic forms and neutral tautomers of cyclic amidines **2** and **3**, DFT-calculated percentage contents of B_1_–B_3_, microscopic basicity parameters for favored protonation sites (indicated in bold blue), and macroscopic basicity parameters (PA_micro_, GB_micro_, PA_macro_, and GB_macro_, in kJ mol^−1^).

Amidines **2** and **3** can be considered as cyclic analogs of acetamidine **1**. They display analogous tautomeric and acid-base equilibria. Only configurational isomerism about the double C=N bond is absent due to the molecule symmetry. Two labile protons can move between three conjugated sites (two N atoms and one methylene C atom) together with the migration of π-electrons. Three potential tautomers (B_1_–B_3_, with B_1_ and B_2_ being structurally identical) are thus possible for **2** and **3** (Figure 5). An intramolecular transfer of a proton from endo C-sp^3^ at the 4-position to endo C-sp^2^ at the 2-position in **2**-B_1_/B_2_ can be neglected because of the low acidity of endo CH in B_1_ and B_2_. The acidity of the exo CH_3_ group in B_1_/B_2_ is considerably stronger than that of the endo CH groups. It is enhanced by both the hyperconjugation of Me with C=N (resonance) and the inductive effect of NH, while that of the endo CH group is influenced by only weaker inductive effects of N atoms.

In the case of aromatic 2-methylimidazole (**3**), the transfer of the labile proton from the amino N atom to the endo C atoms at the 2-, 4-, or 5-position gives three possible CH tautomers of very high relative Gibbs energies (83.4 for C2H and 61.7 kJ mol^−1^ for C4H and C5H tautomers of identical structure), like for neutral unsubstituted imidazole [23]. Our calculations indicate that these neutral CH tautomers, as well as their monoprotonated forms, can be neglected in the tautomeric mixture of **3**. Consequently, for the tautomeric and acid-base equilibria of the tautomeric mixtures of **2** and **3**, we considered three tautomers B_1_–B_3_ for the monoprotonation reaction at the favored protonation sites and one common monoprotonated form (BH^+^), like in acetamidine **1**. The percentage contents of B_1_–B_3_ and their micro- and macroscopic basicity parameters are included in Figure 5.

As could be expected based on the calculations for **1**, the percentage contents of tautomer B_3_ in the tautomeric mixtures of **2** and **3** are extremely small (<1 ppm). Their energetic parameters (*E*, *H*, and *G*) and, consequently, their microscopic basicity parameters (PAs and GBs) are very high in comparison to those of B_1_/B_2_. Although they appear to be superbases, they contribute very little to the equilibrium tautomeric mixtures nor to the estimated macroscopic basicity parameters. The tautomeric mixtures consist mainly of B_1_/B_2_ (100%), and the macroscopic basicity parameters are equal to their microscopic ones.

**Figure 6:**
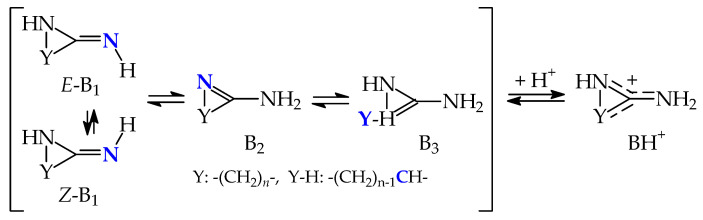
Tautomeric and acid–base equilibria for monoprotonation of cyclic amidines **4**–**7** (site of protonation, the imino N atom in B_1_ and B_2_, and the C atom in the Y group, indicated in bold blue).

Gas-phase tautomeric and acid-base equilibria for cyclic amidines **4**–**7** containing the exo imino N atom (shown in Figure 6) are also analogous to those of acyclic acetamidine **1**. These cyclic systems contain two labile protons and three tautomeric sites. One labile proton in **4**–**7** can move between N atoms together with the migration of π-electrons according to prototropy in the amidine group leading to two different tautomers, i.e., B_1_ and B_2_. The other labile proton can rearrange according to imino-enamino conversion from the ^α^C atom in the Y group bound to the functional amidine C atom to the imino N atom in B_1_ or B_2_ together with the migration of π-electrons leading to the third tautomer, i.e., B_3_. The monoprotonation of B_1_–B_3_ at their favored sites of protonation (imino N in B_1_ and B_2_, and ^α^C in B_3_) gives one common monocation BH^+^. Note that configurational isomerism is possible for B_1_, for which two isomers with the configuration *E* and *Z* about C=N are possible.

Our computational results for cyclic amidines **4**–**7** are included in Table 2. Although the percentage contents of the tautomer B_3_ increase with an increase of C-atoms numbers in the cycle, all B_3_ isomers can be considered as exceptionally rare forms, and they can be neglected in the tautomeric mixture being in equilibrium. Generally, the tautomer B_2_ is favored for cyclic amidines, and B_1_ is a minor form. One exception is **5**, for which B_2_ is a minor form and B_1_ is preferred in the tautomeric mixture. The amounts of the *E*-isomer are higher than those of the *Z*-isomer. The macroscopic basicity parameters are close to the microscopic ones of the favored forms. Bouchoux and Eckert-Maksić [6] investigated only tautomers B_1_ of amidines **4**–**7** at the G4MP2 level, but without an indication of the configuration of C=N, and calculated the following PAs: 915, 978, 1000, and 1008 kJ mol^−1^. These PAs can be considered as microscopic parameters of B_1_, but it is not clear to which isomer, *E* or *Z*, they can be attributed.

### 2.3. Basicity of Tautomeric Cyclic Systems Containing the Guanidine Group

**Figure 7:**
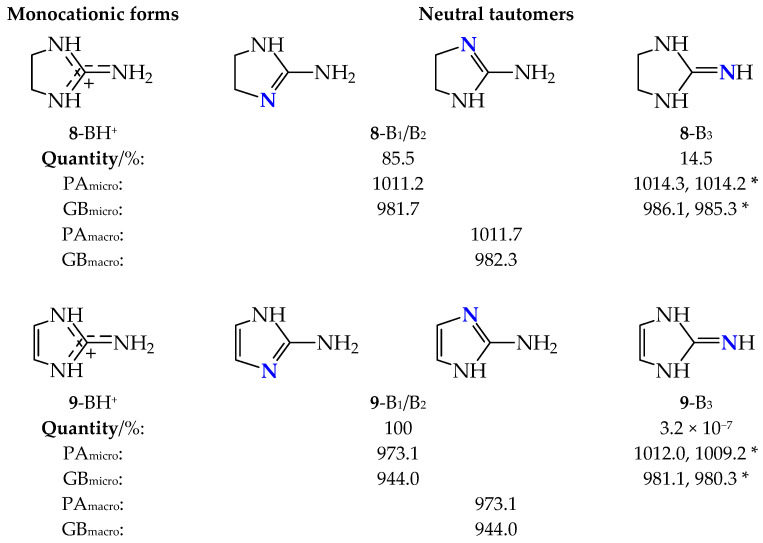
Structures of monocationic forms and neutral tautomers of cyclic guanidines **8** and **9**, DFT-calculated percentage contents of B_1_–B_3_, microscopic basicity parameters for favored protonation sites (indicated in bold blue), and macroscopic basicity parameters (PA_micro_, GB_micro_, PA_macro_, and GB_macro_, in kJ mol^−1^, * indicates PA and GB data taken from Ref. [11]).

Prototropic tautomerism takes also place for the cyclic derivatives **8** and **9**, which can be ranked among the guanidine family. Two labile protons can move between three N atoms, leading to three tautomers B_1_, B_2_, and B_3_ (Figure 7). Two of them (B_1_ and B_2_) possess identical structures completely different from B_3_. Owing to the symmetry of the molecules in B_3_, configurational isomerism about C=N is absent.

Interestingly, the three tautomers (B_1_–B_3_) contribute significantly to the tautomeric mixture of **8** with a strong preponderance of B_1_/B_2_ (86%). For **8**, only tautomer B_3_ was investigated by Koppel and co-workers [11]. Their estimated basicity parameters for **8**-B_3_ are analogous to those for **8**-B_1_/B_2_ at the DFT level. Our calculations indicate that the macroscopic PA and GB for the tautomeric mixture of **8** are close to those of B_1_/B_2_ containing the exo NH_2_ group and having considerably lower energy, enthalpy, and Gibbs energy than B_3_. On the other hand, the tautomer B_3_ in **9** can be considered as an exceptionally rare form. Its amount is very low (3.2 × 10^−7^%). A considerably lower Gibbs energy for aromatic tautomeric forms B_1_/B_2_ also leads to their exceptionally high percentage contents (100%). For this reason, macroscopic PA and GB for the tautomeric mixture of **9** are equal to those of B_1_/B_2_.

It should be mentioned here that the tautomeric protons in **9** can additionally be transferred from the N to C atoms at the 2-, 4-, or 5-position in the imidazole ring, leading to CH tautomers of very high energies, enthalpies, and Gibbs energies relative to that of B_1_/B_2_ (>30 kJ mol^−1^) [24]. These CH tautomers were not considered in the tautomeric mixtures of neutral and monoprotonated forms. Owing to some importance of the CH tautomers with the labile proton at the 4- or 5-position, they will be considered in the future for examination of more complex structures of N-containing superbases.

### 2.4. Basicity of Methyl Derivatives with the Framework of Rare Tautomers of Amidines ***1***–***7***

**Figure 8:**
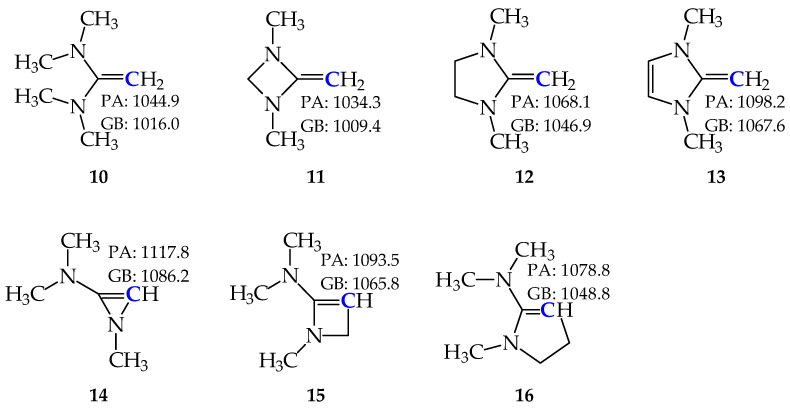
Structures of the strong C-bases studied in this work and their DFT-calculated basicity parameters (PA and GB in kJ mol^−1^) for favored protonation C-sites indicated in bold blue.

For all cyclic and acyclic amidines **1**–**7**, their rare tautomers display considerably higher PAs/GBs than the favored isomers. This theoretical observation encouraged us to study substituted derivatives for which prototropy is absent. In particular, the structural replacement of all labile protons with Me groups excludes tautomerism. Figure 8 summarizes the methyl derivatives (**10**–**16**) investigated in this work. All of them are monoprotonated at the exo C-sp^2^ atom. Thus, all derivatives belong to the family of C-bases. The PA and GB values were computed at the DFT level. They are higher than those of **DMAN**. All of them can be recognized as exceptionally strong C-bases. Their basicity parameters vary in the same range of the PA and GB scales as experimental PAs/GBs for strong organic push-pull N-bases, such as amidines, guanidines, and phosphazenes [2]. This means that after successful syntheses, they could be experimentally investigated and their PAs/GBs determined, for example, by the equilibrium method using an FT-ICR apparatus.

**Figure 9:**
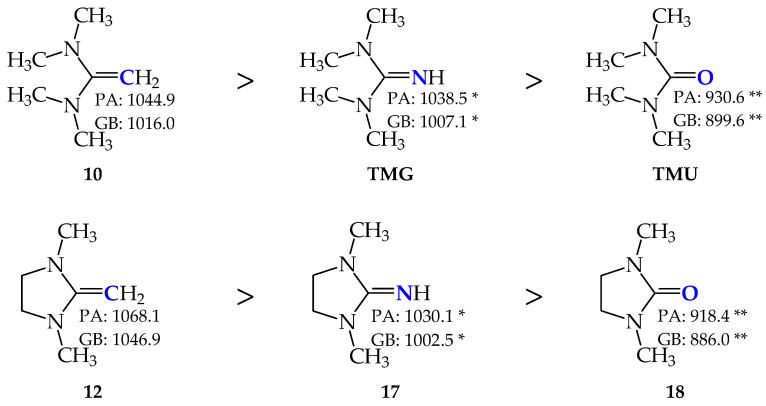
Comparison of gas-phase proton basicity parameters for structurally analogous C-, N-, and O-bases (PA and GB in kJ mol^−1^, * indicates data from Ref. [11] and ** refers to data from Ref. [7]). Favored protonation sites indicated in bold blue.

Remarkably, the DFT-computed PA and GB values for **10** are higher than those of **TMG** (Figure 9) estimated at the same level of theory by Koppel and co-workers [11]. They are also considerably higher than the values obtained experimentally for tetramethylurea (**TMU**, taken from Ref. [7]), indicating a significant increase in the push-pull effect when proceeding from **TMU** to **TMG**, and next to **10**. This increase is in a reverse relation with the electronegativity of the protonation site (C < N < O). The same is true for the DFT-calculated PA/GB of **12** when compared to those of the dimethyl derivative of **8**-B_3_ (**17**) estimated at the same level of theory by Koppel and co-workers [11] and to the experimental data of the corresponding Me derivative of cyclic O-base (1,3-dimethyl-2-imidazolidinone, **18**) taken from Ref. [7].

Although C-bases are structurally analogous to N- and O-bases, protonation sites have different features. For C-bases, when they are protonated, the new σ-bond with the proton is formed from the π-electrons of the C=C double bond, whereas for N- and O-bases, this bond formed from an electron lone pair of the heteroatom. Hence, C-bases can be considered as π-bases, whereas N- and O-bases are n-bases. In the Lewis structure, the pair of π-electrons in C-bases is inside the molecule, whereas the pair of nonbonding electrons in N- and O-bases is outside the molecule. This principal difference seems to play a significant role in the transmission of substituent effects and the basicity of the protonation site.

**Figure 10:**
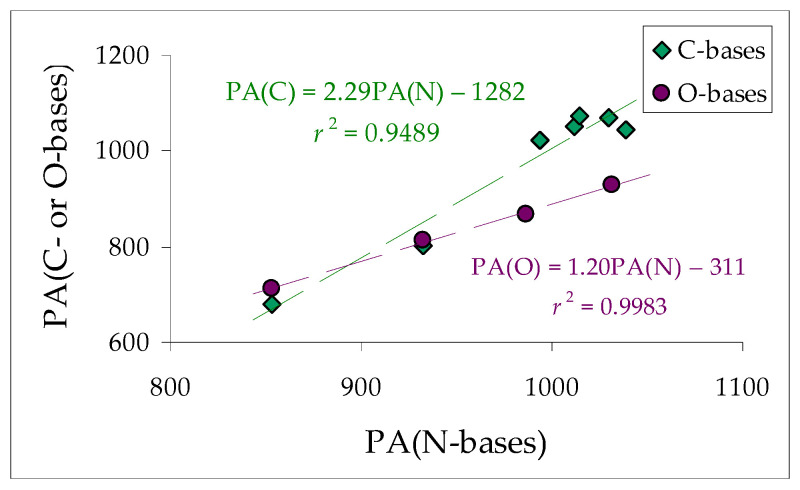
Linear trend between DFT-calculated proton affinities (PA in kJ mol^−1^) of push-pull C- or O-bases and N-bases. PAs data taken from this work and Refs [11,22]. Experimental PAs [7,25] are also included for simple pairs of bases: H_2_C=CH_2_ and H_2_C=NH and also Me_2_C=CH_2_ and Me_2_C=NH. Points for the second linear trend refer to experimental the PAs [7,25] of O- and N-bases.

Figure 10 shows a strong difference in the sensitivity of C-bases on protonation in comparison to N- and O-bases. The slope of the regression line between the PAs of C-bases and the PAs of N-bases is almost twice as high as that between the PAs of O-bases and the PAs of N-bases. Noteworthily, the correlation coefficient between the PAs of O- and N-bases containing protonation sites of similar character is close to unity (*r* = 0.9991), whereas that between the PAs of C- and N-bases, possessing protonation sites of different characters, is considerably lower (*r* = 0.9741). Nevertheless, this indicates a quite good linearity in the transmission of substituent effects in a series of C- and N-bases.

In 2001, Koppel et al. [22] additionally reported DFT-calculated PAs/GBs for numerous organic C-bases. However, some of these compounds are tautomeric systems, particularly those containing the NH_2_ group conjugated with the >C=CH_2_ part. In tautomeric equilibrium, the labile proton(s) at the amino N atom(s) can move to the methylene C atom together with the migration of π-electrons, leading to more stable imino forms. Hence, PAs/GBs proposed for the investigated rare forms of C-bases may not be measured by experimental techniques. This question needs additional investigation in the future for complete tautomeric mixtures of proposed C-forms and their alkyl derivatives.

## 3. Methodology

In this work, the same level of theory was employed to study the prototropic tautomerism and the gas-phase proton basicity for strong push-pull N-bases **1**–**9** as previously chosen by Koppel, Maksić, and their co-workers for superbases without prototropy [3,4]: the DFT (Density Functional Theory) method [26] using the B3LYP (Becke three-parameter hybrid exchange functional and non-local correlation functional of Lee, Yang, and Par) functional [27,28] and the 6-311+G(d,p) basis set [29]. For some derivatives, the Gaussian-*n* methods (G2, G2MP2, and/or G3B3; see the list of abbreviations) [30] were also applied.

The geometries of isolated neutral and monoprotonated forms were optimized without symmetry constraints in their ground states. Their atom coordinates and DFT-calculated electronic energies are summarized in Table S1 (Supplementary Material). Vibrational frequencies and energetic parameters, such as energy (*E*_0_), enthalpy (*H*_298_), entropy (*S*_298_), and Gibbs energy (*G*_298_), were calculated using the same method as that employed for geometry optimization. Indexes 0 and 298 correspond to the temperatures of 0 and 298.15 K. The relative thermochemical parameters for prototropic tautomers (Δ*E*_0_, Δ*H*_298_, *T*Δ*S*_298_, and Δ*G*_298_) and isomeric equilibrium constants (*K*) were also calculated. The Δ*G*_298_ values include variations in the electronic energy, zero-point energy (ZPE), and thermal corrections to the energy and entropy (vibrational, rotational, and translational). The DFT-calculated thermochemical data, such as *H*_298_, *G*_298_, and *S*_298_, are listed in Table S2 (Supplementary Material). The *H*_298_ and *G*_298_ values calculated at the G*n* levels for selected derivatives are given in Table S3 (Supplementary Material). The Δ*H*_298_, Δ*G*_298_, *T*Δ*S*_298_, and *K* values and also the percentage contents calculated for the isomers of neutral tautomeric bases are included in Table S4 (Supplementary Material). For all quantum-chemical calculations, Gaussian 03 programs were used [31].

In the gas phase, the thermochemical basicity parameters are determined without any perturbation from the environment. Hence, according to Brønsted theory, Equilibrium (1) relates conjugate pairs of cationic acid and the corresponding neutral base for each tautomer B_i_ in tautomeric mixtures. The microscopic basicity parameters, proton affinity (PA_micro_), and gas-phase basicity (GB_micro_) for individual B_i_ can be estimated from the thermodynamics of (1) as follows. The PA_micro_ values can be calculated as the enthalpy change in the deprotonation reaction, or conversely, as the negative of the enthalpy change in the reverse protonation reaction, as shown in Equation (2). The GB_micro_ values can be calculated as the corresponding Gibbs energy changes in the same reactions, i.e., deprotonation and protonation, respectively, as shown in Equation (3). In Equation (3), Δ*S* is the entropy of the deprotonation reaction (1) [7,25]. In Equations (2) and (3), *H*_298_(H^+^) = *H*_transl_(H^+^) + *RT* = 5/2*RT* = 6.2 kJ mol^−1^ and *G*_298_(H^+^) = *H*_298_(H^+^) − *TS*_transl_(H^+^) = −26.3 kJ mol^−1^, where *S*_transl_(H^+^) = 108.95 J mol^−1^ K^−1^ at 298.15 K [32,33]. Both PA_micro_ and GB_micro_ correspond to absolute (intrinsic) basicity parameters at 298.15 K. They depend mainly on the structure of B_i_ and B_i_H^+^, the protonation site, and all internal effects.

B_i_H^+^ ⇌ B_i_ + H^+^ (1)

PA_micro_ = Δ*H*(B_i_H^+^ → B_i_ + H^+^) = − Δ*H*(B_i_ + H^+^ → B_i_H^+^) = 
*H*_298_(B_i_) + *H*_298_(H^+^) − *H*_298_(B_i_H^+^) (2)

GB_micro_ = Δ*G*(B_i_H^+^ → B_i_ + H^+^) = − Δ*G*(B_i_ + H^+^ → B_i_H^+^) = 
*G*_298_(B_i_) + *G*_298_(H^+^) − *G*_298_(B_i_H^+^) = PA − *T*Δ*S* (3)

For the tautomeric mixtures containing B_i_ tautomers, for which protonation leads to B_i_H^+^ protonated forms, their acid-base reactions (protonation and deprotonation) can be represented by general Equilibrium (4) [1]. The molar fractions of neutral (*x*_i_) and protonated (*y*_i_) forms in prototropic equilibrium can be estimated according to Equations (5) and (6). The macroscopic basicity parameters, i.e., PA_macro_ and GB_macro_, can be calculated according to Equations (7) and (8). These macroscopic thermodynamic parameters refer to 298.15 K and give some indication of the expected experimental data obtained by the equilibrium method.

[*y*_1_B_1_H^+^ ⇌ *y*_2_B_2_H^+^ ⇌ *y*_3_B_3_H^+^ ⇌ …] ⇌ [*x*_1_B_1_ ⇌ *x*_2_B_2_ ⇌ *x*_3_B_3_ ⇌ …] + H^+^ (4)

*x*_i_ = exp{−Δ*G*_298_(B_i_)/*RT*}/{Σ_1_^n^exp[−Δ*G*_298_(B_i_)/*RT*]} (5)

*y*_i_ = exp{−Δ*G*_298_(B_i_H^+^)/*RT*}/{Σ_1_^n^exp[−Δ*G*_298_(B_i_H^+^)/*RT*]} (6)

PA_macro_ = Σ_1_^n^*x*_i_*H*_298_(B_i_) + *H*_298_(H^+^) − Σ_1_^n^*y*_i_*H*_298_(B_i_H^+^) (7)

GB_macro_ = Σ_1_^n^*x*_i_*G*_298_(B_i_) + *G*_298_(H^+^) − Σ_1_^n^*y*_i_*G*_298_(B_i_H^+^) (8)

For selected methyl-substituted bases derived from rare tautomers, geometries were optimized and frequencies and thermodynamic parameters were estimated at the same DFT(B3LYP)/6-311+G(d,p) level. Gas-phase proton basicity parameters (PAs and GBs) for the favored monoprotonation sites (methylene C atom) in methyl derivatives were calculated analogously as microscopic parameters for individual isomers according to Equations (2) and (3).

It should be mentioned here that the entropy of mixing [34] was not considered for tautomeric bases **1**–**9**, owing to the small number of isomers and their concentrations at equilibrium. Only for one compound (**5**), the relative Gibbs energies (Δ*G*_298_) are lower than 4 kJ.mol^−1^ (Table S4 in the Supplementary Material). For the other derivatives, they are close to or considerably higher than 4 kJ.mol^−1^. The relative entropy terms (*T*Δ*S*_298_) included in Δ*G*_298_ are lower than 4 kJ mol^−1^ for **1**–**9**, like those for other tautomeric systems examined earlier by us (see, for example, Refs [35,36,37]). For derivatives **1**–**9**, isomeric conversions in vacuo seem to be iso-entropic processes, analogously to protonation/deprotonation reactions for organic compounds for which changes in the symmetry do not take place during proton-transfer reactions [7,25].

## 4. Conclusions

Quantum-chemical calculations were applied to different tautomeric forms of organic N-bases. We showed that their rare tautomers always have considerably higher proton affinity and Gibbs energy than the most stable forms. Although they possess exceptionally high microscopic PAs/GBs, experimental determinations of their GBs and PAs by the equilibrium method, using the common techniques employed for the investigation of proton-transfer reactions, are not achievable. Using the equilibrium method, the rare tautomers would be quickly transformed into their favored tautomers, and the measured PAs/GBs would not refer to the basicity of rare tautomers but to those of the favored forms of considerably weaker basicity. According to the Brønsted theory, for tautomeric mixtures in equilibrium, the participation of rare tautomers is exceptionally low, and they do not influence the measured (macroscopic) basicity parameters.

For some tautomeric forms reported in the present study, high basicity is expected based on the calculations performed on the rare tautomers, but due to their small abundance in the tautomeric mixture, this high basicity may be hardly observable. This problem can be solved by substituting the labile proton with methyl groups. The resulting bases, for which prototropy is absent, might be submitted to experimental determination of the gas-phase proton basicity parameters. In this work, we proposed structures of rather simple derivatives (**10**–**16**) containing Me groups instead of tautomeric protons at the amino N atoms. Using DFT calculations, we showed their exceptionally high PAs/GBs for the favored site of monoprotonation, the methylidene C atom, generating a new series of organic C superbases. The simple push-pull C-base (NMe_2_)_2_C=CH_2_ (**10**) is estimated to be a stronger base in the gas phase than (NMe_2_)_2_C=NH. For some cyclic structures, PAs are close to or even higher than 1100 kJ mol^−1^. They can be considered as C-hyperbases like some phosphazenes, polyguanides, and vinamidines [1,2,3,4]. In the future, they may find interesting applications like other small push-pull molecules; see, for example, Refs [38,39,40,41,42,43,44,45,46,47].

## Figures and Tables

**Table 1:** Contents (% at equilibrium) for isomers of acetamidine **1**, their microscopic proton affinities and gas-phase basicities, and macroscopic basicity parameters for the tautomeric mixture (PA_micro_, GB_micro_, PA_macro_, and GB_macro_, respectively, in kJ mol^−1^ at 298.15 K) calculated at the DFT and G*n* levels and compared with other theoretical and experimental data.

Method	Quantity	*E*-B_1_/B_2_	Z-B_1_/B_2_	B_3_	Quantity	Mixture
DFT ^a^	PA_micro_	982.2	986.2	1021.6 1021.7 ^b^	PA_macro_	982.6
	GB_micro_	950.9	995.0	993.0 993.3 ^b^	GB_macro_	951.2
	%	84.2	15.8	3.5 × 10^−6^		
G2 ^a^	PA_micro_	975.8 975.7 ^c^	979.9	1018.5	PA_macro_	976.4
	GB_micro_	945.8	950.1	990.6	GB_macro_	946.4
	%	85.3	14.7	1.2 × 10^−6^		
G2MP2 ^a^	PA_micro_	975.7 975.7 ^c^	979.8	1018.4	PA_macro_	976.3
	GB_micro_	945.6	950.0	990.4	GB_macro_	946.3
	%	85.5	14.5	1.2 × 10^−6^		
G3B3 ^a^	PA_micro_	977.5	981.5	1017.4	PA_macro_	978.1
	GB_micro_	946.8	951.3	989.2	GB_macro_	947.4
	%	86.2	13.8	3.2 × 10^−6^		
G4MP2 ^d^	PA	975.1				
Experiment ^e^					PA_exp_	970.7
					GB_exp_	938.2

^a^ This work. ^b^ Ref. [22]. ^c^ Ref. [9]. ^d^ Ref. [6]. ^e^ Refs. [7,9].

**Table 2:** Percentage contents (% at equilibrium) for isomers of amidines **4**–**7**, their microscopic proton affinities and gas-phase basicities, and macroscopic basicity parameters for the tautomeric mixture (PA_micro_, GB_micro_, PA_macro_, and GB_macro_ in kJ mol^−1^) calculated here at the DFT and/or G*n* levels.

Amidine	Method	Quantity	*E*-B_1_	Z-B_1_	B_2_	B_3_	Quantity	Mixture
**4**	DFT	PA_micro_	923.8	924.7	902.0	1056.9	PA_macro_	902.0
		GB_micro_	893.6	894.4	869.3	1022.2	GB_macro_	869.3
		%	0.0_05_	0.0_04_	99.9_91_	1.6 × 10^−25^		
	G2	PA_micro_	916.3	917.0	894.1	1052.4	PA_macro_	894.1
		GB_micro_	886.6	887.2	862.4	1020.6	GB_macro_	862.4
		%	0.0_06_	0.0_04_	99.9_90_	3.4 × 10^−26^		
	G2MP2	PA_micro_	916.2	916.3	894.8	1055.1	PA_macro_	894.8
		GB_micro_	886.5	887.2	863.1	1019.0	GB_macro_	863.1
		%	0.0_08_	0.0_06_	99.9_86_	4.8 × 10^−26^		
	G3B3	PA_micro_	918.2	918.5	895.2	1052.6	PA_macro_	895.2
		GB_micro_	887.9	888.2	863.1	1018.2	GB_macro_	863.1
		%	0.0_04_	0.0_04_	99.9_92_	6.4 × 10^−26^		
**5**	DFT	PA_micro_	982.6	985.3	990.3	1049.2	PA_macro_	983.5
		GB_micro_	950.5	953.2	958.0	1017.0	GB_macro_	951.4
		%	72.5	23.9	3.6	2.2 × 10^−12^		
	G2	PA_micro_	977.7	980.4	981.3	1042.8	PA_macro_	978.9
		GB_micro_	946.1	948.7	949.2	1010.9	GB_macro_	947.2
		%	61.2	21.3	17.5	2.7 × 10^−10^		
	G2MP2	PA_micro_	977.7	980.4	981.3	1042.8	PA_macro_	978.7
		GB_micro_	946.1	948.7	949.2	1010.9	GB_macro_	947.0
		%	66.5	22.7	10.8	3.1 × 10^−10^		
**6**	DFT	PA_micro_	1005.8	1009.8	999.5	1061.5	PA_macro_	1000.1
		GB_micro_	973.9	977.9	967.4	1029.0	GB_macro_	968.0
		%	6.6	1.3	92.1	1.2 × 10^−9^		
**7**	DFT	PA_micro_	1015.2	1021.0	1010.9	1056.0	PA_macro_	1011.5
		GB_micro_	984.4	989.9	979.3	1021.13	GB_macro_	980.0
		%	11.4	1.2	87.4	3.8 × 10^−6^		

## Data Availability

The original contributions presented in this study are included in the article/Supplementary Material. Further inquiries can be directed to the corresponding author.

## Supplementary Materials

The following supporting information can be downloaded at https://www.mdpi.com/article/10.3390/molecules30030474/s1, Table S1. DFT-calculated atom coordinates and electronic energies (*E* in Hartree) for investigated derivatives. Table S2. DFT-calculated enthalpies (*H* in Hartree) and Gibbs energies (*G* in Hartree) at 298.15 K with zero-point energies and thermal corrections included, and entropies (*S* in cal mol^−1^ K^−1^) for neutral and monoprotonated forms of **1**–**16.** Table S3. G*n*-calculated enthalpies (*H* in Hartree) and Gibbs energies (*G* in Hartree) at 298.15 K for isomers of **1**, **4**, and **5.** Table S4. DFT-calculated relative enthalpies, entropy terms, Gibbs energies (Δ*H*_298_, *T*Δ*S*_298_, and Δ*G*_298_ in kJ mol^−1^, respectively), tautomeric equilibrium constants (*K*), and percentage contents (%) for neutral tautomeric bases **1**–**9.**

## Abbreviations

A	acceptor
B	neutral base
B_i_	tautomer of B
**BEMP**	2-*tert*-butylimino-2-diethylamino-1,3-dimethylperhydro-1,3,2-diazaphosporine
BH^+^	conjugate acid of B
Bu	buthyl group
D	donor
**DBD**	1,5-diazabicyclo[4.4.0]dec-5-ene
**DBN**	1,5-diazabicyclo[4.3.0]non-5-ene
**DBU**	1,8-diazabicyclo[5.4.0]undec-7-ene
DFT	density functional theory
**DMAN**	1,8-bis(dimethylamino)naphthalene
*E*	energy
Et	ethyl group
FT-ICR	Fourier transform mode of ICR
*G*	Gibbs energy
G2	Gaussian-2 theory
G2MP2	MP2 variant of Gaussian-2 theory
G3B3	Gaussian-3 theory with B3LYP structures and frequencies
G4MP2	MP2 variant of Gaussian-4 theory
G*n*	Gaussian-*n* theory
GB	gas-phase basicity
*H*	enthalpy
*K*	equilibrium constant
Me	methyl group
MP2	second-order Møller-Plesset (perturbation theory)
**MTBD**	7-methyl-1,5,7-triazabicyclo[4.4.0]dec-7,9-ene
PA	proton affinity
*R*	molar gas constant
*S*	entropy
*T*	temperature
**TMG**	N^1^,N^1^,N^3^,N^3^-tetramethylguanidine
**TMU**	N,N,N’,N^’^-tetramethylurea
*x*_i_	molar fraction of B isomer
*y*_i_	molar fraction of BH^+^ isomer
ZPE	zero-point energy
