# Supplementary material for: Potential Push-Pull Carbon Superbases Based on Methyl Substitution of Rare Tautomers of Imines
Source: Molecules. 2025 Jan 22;30(3):474. doi: 10.3390/molecules30030474 (PMC11821125; doi:10.3390/molecules30030474)
Supplement: Supplementary file 1 [file molecules-30-00474-s001.zip › molecules-3409195-supplementary.pdf]

Supplementary Material

Potential Push-Pull Carbon Superbases Based on Methyl Substitution of Rare Tautomers of Imines

Ewa Daniela Raczyńska,[1*] Jean-François Gal,[2] Pierre-Charles Maria[2]

[1] Department of Chemistry, Warsaw University of Life Sciences (SGGW), ul. Nowoursynowska 159c, 02-776 Warsaw, Poland

[2] Institut de Chimie de Nice, UMR 7272, Université Côte d'Azur, Parc Valrose, 06108 Nice, France

**Contents**	**Page**
Atom coordinates and DFT-calculated electronic energies for compounds **1–16** (Table S1)	S2
DFT-calculated enthalpies, Gibbs energies, and entropies for neutral and monoprotonated forms of **1–16** (Table S2)	S12
G*n*-calculated enthalpies and Gibbs energies for **1**, **4**, and **5** (Table S3)	S14
DFT-calculated relative enthalpies, entropy terms, Gibbs energies, tautomeric equilibrium constants, and amounts for **1-9** (Table S4)	S15

Table S1. DFT-calculated atom coordinates and electronic energies (*E* in Hartree) for investigated derivatives

1-*E*-B$_1$/B$_2$

E = -189.391198

C	-2.611266	-0.283679	0.042820
C	-2.672170	1.229541	0.053917
N	-3.729543	1.948966	0.053872
N	-1.435858	1.845688	0.005022
H	-3.613227	-0.710712	0.076073
H	-2.106975	-0.638344	-0.860513
H	-2.049651	-0.657398	0.905141
H	-1.443663	2.841453	0.172144
H	-0.634455	1.350130	0.361139
H	-4.563652	1.368226	0.027355

1-Z-B$_1$/B$_2$

E = -189.389415

C	-2.576920	-0.249348	0.038338
C	-2.639537	1.257921	0.038847
N	-1.596869	1.936471	0.333235
N	-3.897411	1.776119	-0.241304
H	-3.297167	-0.665699	0.748868
H	-2.814787	-0.649478	-0.952907
H	-1.574071	-0.565022	0.316264
H	-1.777854	2.942145	0.300364
H	-3.965160	2.758852	-0.459241
H	-4.561994	1.190300	-0.722673

1-B$_3$

E = -189.376310

C	-2.389972	0.473518	-1.073205
C	-2.690654	1.343479	-0.092878
N	-3.971893	1.513813	0.450394
N	-1.763931	2.198865	0.519682
H	-3.106371	-0.267276	-1.401823
H	-1.423754	0.491506	-1.559095
H	-4.659859	0.871550	0.083795
H	-3.981600	1.536749	1.463089
H	-0.839425	2.156748	0.115072
H	-2.091991	3.153177	0.608209

1-BH$^+$

E = -189.776859

C	-2.334829	0.333329	-1.115601
C	-2.693482	1.355000	-0.084427
N	-3.911386	1.361663	0.427037
N	-1.796201	2.253948	0.278539
H	-2.900941	-0.586640	-0.970175
H	-1.270445	0.100790	-1.084046
H	-4.586770	0.666092	0.143597
H	-4.221845	2.048779	1.102144
H	-0.867166	2.235131	-0.117491
H	-1.991685	2.989597	0.945537
H	-2.572360	0.732511	-2.106925

2-B$_1$/B$_2$

E = -266.814403

N	-1.627441	-0.081111	-0.039360
C	-0.388732	-0.389505	0.054035
N	0.498474	0.679967	0.215536
C	-0.252944	1.896172	-0.133154
C	-1.705950	1.388625	0.051834
C	0.135000	-1.790738	0.064662
H	1.434383	0.574331	-0.150981
H	-0.061311	2.199187	-1.170951
H	0.000733	2.728057	0.525991
H	-2.395370	1.786968	-0.694949
H	-2.096415	1.659526	1.040012
H	0.642293	-2.000928	1.010788
H	-0.691523	-2.487643	-0.061347
H	0.859373	-1.947290	-0.741404

2-B$_3$

E = -266.795751

N	-1.756429	0.102479	-0.329368
C	-0.523814	-0.485626	-0.003700
N	0.347203	0.564068	0.329077
C	-0.255930	1.838291	-0.066467
C	-1.745131	1.508875	0.077808
C	-0.233905	-1.798723	-0.009398
H	-2.574022	-0.440369	-0.094143
H	1.316922	0.417881	0.090857
H	-0.023771	2.108440	-1.106252
H	0.060807	2.651031	0.589914
H	-2.375816	2.118111	-0.572403
H	-2.067552	1.647551	1.119367
H	-0.982840	-2.530268	-0.277771
H	0.753908	-2.149132	0.254279

2-BH$^+$

E = -267.208051

N	-1.584122	-0.019491	0.133558
C	-0.325257	-0.403402	0.008887
N	0.469229	0.639793	-0.159025
C	-0.270028	1.920144	-0.152638
C	-1.736807	1.449333	0.062380
C	0.138159	-1.814888	0.080897
H	1.468745	0.565177	-0.279372
H	-0.131739	2.438313	-1.101634
H	0.086765	2.557192	0.656686
H	-2.389127	1.719772	-0.767915
H	-2.165042	1.828516	0.990359
H	0.367207	-2.066336	1.121012
H	-0.634844	-2.496169	-0.275006
H	1.040328	-1.958480	-0.513924
H	-2.351548	-0.661354	0.268305

3-B$_1$/B$_2$

E = -265.615283

N	-1.189038	-0.710296	0.061500
C	-1.201447	0.658912	-0.011668
N	0.025031	1.129832	-0.084428
C	0.859202	0.032429	-0.056655
C	0.130969	-1.121642	0.034075
C	-2.454454	1.470512	-0.003062
H	1.931527	0.139389	-0.104032
H	0.411276	-2.160658	0.078878
H	-3.120838	1.198701	-0.828005
H	-2.186305	2.520868	-0.109175
H	-1.994810	-1.311910	0.124508
H	-3.010034	1.349682	0.932695

3-B$_3$

E = -265.572573292

N	-0.098810	-1.096943	-0.038654
C	0.768311	0.000000	-0.000005
N	-0.098810	1.096941	0.038633
C	-1.434701	0.672774	0.001409
C	-1.434701	-0.672774	-0.001397
C	2.121198	0.000000	0.000006
H	-2.254522	1.372297	-0.001084
H	-2.254524	-1.372294	0.001092
H	2.673917	-0.927818	-0.056778
H	2.673917	0.927817	0.056792
H	0.211958	2.028730	-0.174261
H	0.211955	-2.028717	0.174315

3-BH$^+$

E = -265.996119

N	-1.179332	-0.689300	-0.045147
C	-1.246874	0.649147	-0.007064
N	0.022585	1.077400	0.043765
C	0.901847	0.005484	0.040860
C	0.139955	-1.114446	-0.015466
C	-2.474982	1.483615	0.014452
H	1.969920	0.134518	0.072839
H	0.412052	-2.155452	-0.042309
H	-3.284306	0.991120	-0.526234
H	-2.290440	2.451935	-0.452643
H	-1.987061	-1.297446	-0.088184
H	-2.803279	1.655095	1.044211
H	0.291795	2.052231	0.080440

4-*E*-B$_1$

E = -188.104421

N	-1.448593	-1.110100	0.831998
C	-1.675583	0.386180	0.888699
C	-1.956117	-0.469514	-0.269578
N	-2.409079	-0.727219	-1.413969
H	-2.067461	-1.713898	1.365101
H	-0.763206	0.965902	0.977050
H	-2.528466	0.765744	1.446604
H	-2.664154	0.131176	-1.901525

4-*Z*-B$_1$

E = -188.103992

N	-1.429898	-1.134511	0.822192
C	-1.698023	0.365593	0.855577
C	-1.988946	-0.496140	-0.275558
N	-2.459830	-0.615631	-1.434157
H	-2.044843	-1.719282	1.381733
H	-0.798205	0.968578	0.903106
H	-2.539433	0.728518	1.440214
H	-2.471913	-1.595394	-1.726757

4-B$_2$

E = -188.112984

N	-1.544840	-1.145639	0.771489
C	-1.706231	0.398917	0.854648
C	-2.000072	-0.513857	-0.218896
N	-2.497388	-0.691234	-1.456678
H	-0.791021	0.987625	0.846437
H	-2.499629	0.772692	1.498857
H	-2.397565	-1.594655	-1.896594
H	-2.608074	0.107240	-2.058684

4-B$_3$

E = -188.052708

N	-0.926268	-0.204647	-0.779773
C	-0.775678	1.049449	-0.126675
N	-1.559894	2.129752	-0.007801
C	0.324013	0.372949	0.044996
H	-2.445842	2.122623	-0.488547
H	1.359742	0.352075	-0.237932
H	-1.144957	3.029047	0.182263
H	-1.404395	-0.867958	-0.166301

4-BH+

E = -188.467385

N	-1.596428	-1.065678	0.799373
C	-1.750707	0.423117	0.852876
C	-2.045097	-0.487914	-0.260831
N	-2.517848	-0.697201	-1.448077
H	-1.783401	-1.881023	1.374518
H	-0.820193	0.978972	0.878203
H	-2.595464	0.831975	1.401447
H	-2.638047	-1.634819	-1.817626
H	-2.750865	0.083582	-2.050404

5-*E*-B1

E = -227.464017

N	-2.288613	-1.274073	-0.115805
C	-2.307631	0.101464	0.032220
N	-3.296817	0.885042	0.143347
H	-3.024166	-1.918061	0.140881
H	-2.992545	1.852573	0.224784
C	-0.767842	0.153588	0.044613
C	-0.835022	-1.401863	0.076226
H	-0.335115	0.628476	0.925336
H	-0.327576	0.576078	-0.859286
H	-0.544013	-1.834302	1.038418
H	-0.323372	-1.926264	-0.733665

5-*Z*-B1

E = -227.462847

N	-2.285766	-1.301510	-0.151479
C	-2.309109	0.085257	0.000316
N	-3.196498	0.983683	0.084343
H	-3.004566	-1.958094	0.122193
C	-0.783191	0.144244	0.052449
C	-0.830865	-1.412327	0.073939
H	-0.383832	0.621843	0.946569
H	-0.325258	0.581035	-0.835224
H	-0.559533	-1.849269	1.039368
H	-0.298650	-1.927684	-0.728043
H	-4.140822	0.597770	0.040920

5-B2

E = -227.460984

N	-2.341645	-1.279765	-0.118289
C	-2.240457	0.003231	0.001833
N	-3.236289	0.937822	0.069251
C	-0.746557	0.139926	0.133164
C	-0.853955	-1.411062	-0.004184
H	-0.385055	0.517566	1.092607
H	-0.236612	0.662235	-0.680518
H	-0.540193	-1.977812	0.876165
H	-0.391590	-1.839827	-0.896929
H	-4.159128	0.626621	-0.200989
H	-3.010140	1.887025	-0.181789

5-B3

E = -227.438590

N	-0.806445	-0.674553	-0.204168
C	-0.695498	0.727877	0.019373
N	-1.791285	1.555581	0.159378
C	0.705820	-0.714454	-0.084730
C	0.653010	0.795428	0.051724
H	-1.301941	-1.188370	0.523106
H	-2.565537	1.320077	-0.448437
H	-1.580965	2.543772	0.146601
H	1.177811	-1.124134	-0.981891
H	1.034187	-1.275003	0.796596
H	1.387701	1.583469	0.022468

5-BH+

E = -227.849962

N	-2.301800	-1.252937	0.051883
C	-2.275893	0.066474	0.047732
N	-3.239680	0.957506	0.021658
H	-3.063641	-1.922133	0.036298
H	-3.019774	1.944149	0.022551
C	-0.770385	0.155672	0.083634
C	-0.822002	-1.407650	0.087617
H	-0.367944	0.615299	0.986657
H	-0.323939	0.610102	-0.801093
H	-0.467128	-1.889164	0.997037
H	-0.424601	-1.894934	-0.800919
H	-4.218582	0.700427	-0.000925

6-*E*-B$_1$

E = -266.820657

N	-2.197770	-1.276526	0.399714
C	-2.293278	0.088785	0.256743
N	-3.376462	0.734696	0.463664
H	-3.047613	-1.815143	0.474955
C	-0.918594	0.580354	-0.195276
C	-0.960906	-1.835650	-0.127492
H	-0.602662	1.458139	0.370766
H	-0.989069	0.873335	-1.248372
H	-1.067384	-2.157894	-1.173646
H	-0.633347	-2.697109	0.459268
H	-3.231227	1.733299	0.334995
C	0.003970	-0.638984	-0.012495
H	0.449611	-0.625735	0.985155
H	0.812351	-0.685217	-0.743678

6-*Z*-B$_1$

E = -266.818969

N	-2.241033	-1.340693	-0.001013
C	-2.313772	0.036572	0.123495
N	-3.336469	0.793580	0.230238
H	-3.037664	-1.913198	0.234226
C	-0.883879	0.554053	0.114096
C	-0.879958	-1.862000	0.103374
H	-0.628758	0.860433	1.133968
H	-0.792332	1.432855	-0.523417
H	-0.740803	-2.732667	-0.541894
H	-0.634399	-2.157671	1.133284
C	-0.040279	-0.649738	-0.341270
H	0.963969	-0.663135	0.084443
H	0.054009	-0.652712	-1.430244
H	-4.206351	0.258383	0.241955

6-B$_2$

E = -266.822750

N	-2.283972	-1.326490	0.229646
C	-2.243251	-0.049127	0.229753
N	-3.350691	0.744378	0.429986
C	-0.863737	0.576106	0.107022
C	-0.911908	-1.838069	0.133945
H	-0.562223	0.982989	1.079284
H	-0.833807	1.400594	-0.611530
H	-0.870831	-2.677034	-0.565701
H	-0.604091	-2.226140	1.113509
C	-0.024411	-0.645067	-0.311250
H	0.969578	-0.658739	0.139462
H	0.102479	-0.658030	-1.396941
H	-4.239065	0.266854	0.372312
H	-3.338740	1.669684	0.030283

6-B$_3$

E = -266.799093

N	-3.794854	0.320788	0.652537
C	-2.917808	1.173506	-0.012139
C	-3.178241	2.166283	-0.885261
N	-1.557290	1.119564	0.350878
H	-4.719374	0.289699	0.247896
C	-1.896840	2.928451	-1.146791
C	-0.832242	1.919999	-0.659303
H	-1.846852	3.856718	-0.559145
H	-1.741529	3.201320	-2.194315
H	0.046738	2.393641	-0.218859
H	-0.509719	1.286539	-1.497379
H	-3.440197	-0.609054	0.833456
H	-4.162305	2.478864	-1.202796
H	-1.185777	0.188923	0.501303

6-BH$^+$

E = -267.215488

N	-2.296778	0.119188	0.137250
C	-2.243416	1.582335	0.372663
C	-0.815255	1.933776	-0.085171
C	-0.032894	0.608569	0.050197
C	-1.114560	-0.433482	-0.025882
N	-0.891836	-1.723717	-0.205947
H	-2.413287	1.780374	1.433694
H	-3.020175	2.078229	-0.208148
H	-0.380805	2.734481	0.510387
H	-0.825717	2.255297	-1.127586
H	0.459556	0.512578	1.024804
H	0.727556	0.462432	-0.718579
H	0.049602	-2.062552	-0.340745
H	-3.163522	-0.403632	0.178045
H	-1.632928	-2.411746	-0.222132

7-*E*-B$_1$

E = -306.147278

N	-2.197912	-1.100181	0.167184
C	-2.230635	0.275610	0.036105
N	-3.266982	0.822690	-0.484222
H	-2.998913	-1.534172	-0.274503
C	-1.044289	1.014857	0.636094
C	-0.954452	-1.872211	0.117466
H	-1.249152	1.137464	1.706898
H	-0.998636	2.015960	0.201578
H	-0.618056	-2.008983	-0.922632
H	-1.156961	-2.865990	0.525080
H	-3.198325	1.837271	-0.443213
C	0.289597	0.274500	0.460590
C	0.149470	-1.181301	0.913594
H	1.075808	0.784341	1.024852
H	0.589433	0.300725	-0.594067
H	-0.093449	-1.215065	1.981720
H	1.088194	-1.725454	0.775064

7-Z-B$_1$

E = -306.144880

N	-2.197159	-1.124285	0.173881
C	-2.247319	0.260593	0.077582
N	-3.238480	0.942188	-0.364280
H	-2.984124	-1.590602	-0.258270
C	-1.055651	0.998805	0.657070
C	-0.942708	-1.887078	0.124592
H	-1.243436	1.112452	1.732043
H	-1.050047	2.000376	0.227419
H	-0.613015	-2.024612	-0.916828
H	-1.135838	-2.880346	0.538487
C	0.279362	0.273003	0.452696
C	0.160741	-1.183271	0.909008
H	1.071287	0.789039	1.002847
H	0.561085	0.302433	-0.607091
H	-0.071541	-1.217573	1.979483
H	1.101532	-1.721764	0.761946
H	-3.994090	0.338613	-0.697516

7-B$_2$

E = -306.148183

N	-2.145272	-1.065706	-0.226116
C	-2.145314	0.195372	-0.037985
N	-3.271136	0.928773	-0.392082
C	-0.999051	1.032604	0.509714
C	-0.944275	-1.847415	0.049325
H	-1.249508	1.317330	1.540358
H	-0.931367	1.963237	-0.062174
H	-0.483587	-2.107089	-0.914470
H	-1.263878	-2.797159	0.490082
H	-3.431824	1.795581	0.098662
C	0.328420	0.267990	0.483264
C	0.094176	-1.169825	0.950342
H	1.069795	0.776128	1.106263
H	0.724866	0.258518	-0.538774
H	-0.265887	-1.162245	1.986699
H	1.025721	-1.744453	0.940079
H	-4.099458	0.370760	-0.547087

7-B$_3$

E = -306.130848

N	-3.688904	0.333877	0.650698
C	-2.736680	1.181498	0.045763
C	-3.038212	2.188543	-0.799613
N	-1.435037	0.941784	0.470663
H	-4.633810	0.556226	0.367253
C	-1.997629	3.160709	-1.303440
H	-2.345238	4.194256	-1.182367
H	-1.843840	3.026807	-2.383430
H	-3.506635	-0.651999	0.493982
H	-4.067139	2.312944	-1.117743
H	-1.266485	0.075309	0.956100
C	-0.353506	1.511251	-0.320140
C	-0.656692	2.992923	-0.566247
H	-0.241127	0.992959	-1.285965
H	0.580567	1.392923	0.234354
H	0.156985	3.450665	-1.136509
H	-0.702039	3.495855	0.404621

7-BH$^+$

E = -306.545428

N	-2.125683	-1.047388	-0.081466
C	-2.188389	0.257316	0.079870
N	-3.313575	0.922823	-0.166455
H	-2.950290	-1.528547	-0.423388
C	-0.994723	1.029354	0.574128
C	-0.921357	-1.896465	0.114998
H	-1.193041	1.262549	1.627852
H	-0.958002	1.985343	0.045090
H	-0.539908	-2.157914	-0.876183
H	-1.256059	-2.813531	0.601608
H	-3.348595	1.923189	-0.037927
C	0.322396	0.255890	0.438968
C	0.137973	-1.177507	0.940901
H	1.095881	0.771213	1.009745
H	0.648047	0.252746	-0.606006
H	-0.151090	-1.172457	1.996915
H	1.070392	-1.740246	0.868446
H	-4.169428	0.470151	-0.457576

8- B_1/B_2

E = -282.868440

N	-2.041363	-0.145891	-0.138294
C	-0.730726	-0.581410	0.103488
N	0.182950	0.312341	0.159421
N	-0.524773	-1.938107	0.276828
C	-2.010809	1.312439	0.084815
C	-0.489078	1.605200	-0.033565
H	0.454230	-2.188012	0.256379
H	-2.389985	1.565134	1.082438
H	-2.609426	1.840206	-0.659508
H	-0.148037	2.338776	0.700957
H	-0.240326	1.997434	-1.026912
H	-2.764411	-0.657643	0.351213
H	-1.095046	-2.534477	-0.306840

8-B_3

E = -282.867306

N	-2.007638	-0.097749	-0.211065
C	-0.751585	-0.668632	-0.039474
N	0.144278	0.392582	0.156974
N	-0.546617	-1.929413	-0.052538
C	-1.981012	1.311343	0.166538
C	-0.517812	1.664483	-0.143266
H	0.437277	-2.148696	0.081689
H	-2.195136	1.462179	1.233593
H	-2.685916	1.901630	-0.421432
H	-0.139974	2.472830	0.485076
H	-0.406544	1.949715	-1.197648
H	1.086274	0.251361	-0.176410
H	-2.791424	-0.679944	0.043052

8-BH^+

E = -283.264929

N	-1.991584	-0.127021	-0.033102
C	-0.753628	-0.621943	0.072566
N	0.144178	0.365254	0.162184
N	-0.455798	-1.919673	0.086630
C	-1.988308	1.338553	0.156740
C	-0.496844	1.678521	-0.057766
H	0.498096	-2.238326	0.158991
H	-2.323781	1.587080	1.166197
H	-2.636622	1.823583	-0.570611
H	-0.128304	2.412539	0.656378
H	-0.298523	2.027611	-1.073698
H	-1.174975	-2.623746	0.025354
H	1.135702	0.217102	0.044840
H	-2.819857	-0.690193	0.091957

9-B_1/B_2

E = -281.662148

N	-1.967642	-0.249926	0.243415
C	-0.758881	-0.666829	-0.048218
N	0.143704	0.359810	-0.057242
N	-0.397003	-1.968764	-0.390844
C	-1.852132	1.118017	0.419176
C	-0.560753	1.522010	0.244863
H	0.357849	-2.351689	0.165886
H	-2.708162	1.722540	0.674013
H	-0.078234	2.481689	0.317725
H	1.101365	0.303794	-0.364213
H	-1.204632	-2.577692	-0.353780

9-B_3

E = -281.646505

N	-1.960333	-0.171458	0.325179
C	-0.732949	-0.748260	0.030384
N	0.124233	0.351515	-0.021584
N	-0.545694	-2.008205	-0.134739
C	-1.856924	1.213287	0.447368
C	-0.568258	1.542139	0.233630
H	0.424301	-2.224951	-0.343063
H	-2.704100	1.836291	0.673679
H	-0.082273	2.501727	0.238922
H	1.106575	0.275305	-0.217406
H	-2.790217	-0.729382	0.427670

9-BH^+

E = -282.043661

N	-1.986274	-0.109381	-0.028719
C	-0.752283	-0.645674	-0.008959
N	0.114074	0.380419	0.075499
N	-0.445769	-1.948143	-0.062998
C	-1.897340	1.286674	0.045079
C	-0.588386	1.591979	0.110066
H	-2.773235	1.911189	0.043420
H	-0.082094	2.538768	0.176892
H	-1.162448	-2.653736	-0.125768
H	1.119632	0.290252	0.108887
H	-2.846910	-0.634931	-0.087721
H	0.511505	-2.262915	-0.040738

10

E = -346.631455

C	-2.384910	0.459046	-1.089115
C	-2.686784	1.332349	-0.105300
N	-3.943357	1.386120	0.531163
N	-1.779009	2.287641	0.395015
H	-3.048699	-0.352834	-1.346222
H	-1.468711	0.540727	-1.654603
C	-5.039574	0.663329	-0.088784
C	-3.951387	1.285278	1.990789
C	-0.381128	2.137716	0.031832
C	-2.218504	3.683201	0.393931
H	-4.961841	-0.429864	0.034443
H	-5.078312	0.888718	-1.155126
H	-5.977783	0.985075	0.370614
H	-3.111734	1.837791	2.407067
H	-3.877191	0.238154	2.326837
H	-4.882085	1.705508	2.381862
H	-0.174848	2.409308	-1.016970
H	-0.063826	1.106403	0.190263
H	0.220306	2.790242	0.670023
H	-3.269797	3.742970	0.667399
H	-2.091770	4.149055	-0.596937
H	-1.630055	4.253095	1.118278

10H+

E = -347.041848

C	-2.399724	0.369087	-1.295360
C	-2.685931	1.313295	-0.159953
N	-3.836376	1.175903	0.505332
N	-1.811105	2.281096	0.136876
H	-2.692973	-0.651632	-1.052136
H	-1.351606	0.376179	-1.576103
C	-4.941247	0.354248	-0.021570
C	-4.040120	1.617354	1.897057
C	-0.396711	2.228800	-0.284288
C	-2.203919	3.581744	0.711856
H	-4.884046	-0.667761	0.363378
H	-4.939701	0.337863	-1.107784
H	-5.879783	0.799944	0.308223
H	-3.087596	1.831149	2.373382
H	-4.513261	0.793933	2.434634
H	-4.692737	2.490363	1.957076
H	-0.263123	2.655863	-1.281602
H	-0.014285	1.211539	-0.257918
H	0.181245	2.817335	0.427484
H	-3.278931	3.720934	0.645138
H	-1.722728	4.359816	0.116496
H	-1.876750	3.686296	1.748042
H	-2.965591	0.686363	-2.177719

11

E = -306.075445

C	-3.935840	0.046434	-0.047708
C	-2.964399	0.966489	-0.047626
N	-2.902733	2.317238	-0.465669
N	-1.612331	0.954694	0.370535
H	-4.918525	0.280670	-0.431957
H	-3.755335	-0.947552	0.336474
C	-3.870208	3.317933	-0.053809
C	-0.665602	-0.065651	-0.041327
H	-3.918337	3.438707	1.039546
H	-3.616428	4.278870	-0.507007
H	-4.859468	3.031628	-0.413532
H	-0.547524	-0.120174	-1.134676
H	0.307655	0.135546	0.411973
H	-1.005227	-1.037937	0.318284
C	-1.490160	2.362747	-0.047503
H	-1.275988	3.051731	0.780292
H	-0.790492	2.539266	-0.875221

11H+

E = -306.481201

C	-3.967276	0.024789	-0.036361
C	-2.891946	1.035005	-0.047778
N	-2.915749	2.362089	-0.237593
N	-1.569554	0.938733	0.152017
H	-4.792322	0.336871	-0.677592
H	-3.594577	-0.941416	-0.378931
C	-3.943597	3.383306	-0.091688
C	-0.598865	-0.137923	0.011661
H	-3.997006	3.749210	0.937072
H	-3.723376	4.211441	-0.764905
H	-4.905775	2.954960	-0.372130
H	-0.215662	-0.195961	-1.010453
H	0.226043	0.032592	0.703210
H	-1.078385	-1.080477	0.273941
H	-4.348542	-0.098524	0.982731
C	-1.449690	2.402503	-0.033638
H	-1.135838	2.938250	0.862988
H	-0.886223	2.699663	-0.918893

12

E = -345.432582

C	-3.775249	0.277064	0.476238
C	-2.849854	1.098688	-0.062294
N	-3.098491	2.293435	-0.752269
N	-1.459999	0.917185	-0.036447
H	-4.824708	0.526685	0.452875
C	-4.301679	3.053593	-0.488770
C	-0.897738	-0.412029	0.073039
H	-4.341797	3.438252	0.542473
H	-5.183242	2.433904	-0.660336
H	-1.138507	-1.046078	-0.794743
H	0.187873	-0.332597	0.160862
H	-1.268593	-0.904314	0.973440
H	-3.491421	-0.652331	0.945159
C	-1.840197	3.014445	-0.916084
C	-0.843523	1.860713	-0.963651
H	-1.630702	3.674971	-0.058443
H	-1.847084	3.615757	-1.828183
H	0.158012	2.142512	-0.630385
H	-0.773889	1.448016	-1.983730
H	-4.348222	3.896711	-1.181271

12H+

E = -345.851732

C	-3.763973	0.206086	0.582652
C	-2.795200	1.109314	-0.100662
N	-3.039663	2.368232	-0.450501
N	-1.562025	0.733917	-0.427581
H	-4.747979	0.657373	0.670187
H	-3.865122	-0.726035	0.022845
C	-4.295199	3.110724	-0.380048
C	-0.892148	-0.516512	-0.085482
H	-4.977862	2.652390	0.330444
H	-4.083109	4.126331	-0.042930
H	-4.770317	3.153725	-1.363964
H	-0.378055	-0.901733	-0.967623
H	-0.159432	-0.350340	0.709312
H	-1.614832	-1.258117	0.246404
H	-3.403851	-0.039128	1.585135
C	-1.889246	2.938097	-1.184449
C	-0.802524	1.862150	-1.002500
H	-1.611599	3.902491	-0.758069
H	-2.165080	3.085033	-2.232331
H	-0.019241	2.168637	-0.303569
H	-0.335220	1.564515	-1.941729

13

E = -344.213323

C	-3.663460	0.402902	0.791228
C	-2.842322	1.099071	-0.040801
N	-3.084501	2.332176	-0.639415
N	-1.573210	0.736589	-0.482953
H	-4.632848	0.789989	1.063952
C	-4.303798	3.077454	-0.448363
C	-0.922992	-0.491198	-0.097746
H	-4.449644	3.323852	0.609522
H	-4.250151	4.003828	-1.020136
H	-5.171453	2.501777	-0.790235
H	0.056608	-0.541575	-0.572945
H	-0.790600	-0.539080	0.989123
H	-1.512149	-1.360565	-0.411115
H	-3.362488	-0.550877	1.195764
C	-1.988876	2.702733	-1.421806
C	-1.064156	1.726548	-1.325977
H	-1.971967	3.626911	-1.972762
H	-0.091253	1.641556	-1.777836

13H+

E = -344.644319

C	-3.774672	0.233319	0.671209
C	-2.835247	1.060689	-0.129191
N	-3.088873	2.276286	-0.649087
N	-1.585008	0.719598	-0.493849
H	-4.453228	0.863286	1.246698
H	-4.378296	-0.410603	0.022749
C	-4.341731	3.030495	-0.494527
C	-0.899473	-0.531972	-0.138949
H	-4.476200	3.328606	0.545570
H	-4.280497	3.921486	-1.114986
H	-5.186137	2.426434	-0.824518
H	0.046941	-0.564231	-0.673630
H	-0.704844	-0.564075	0.933371
H	-1.503777	-1.387933	-0.437071
H	-3.232304	-0.404480	1.369363
C	-1.978234	2.712596	-1.349145
C	-1.037974	1.739305	-1.252055
H	-1.954875	3.665477	-1.849082
H	-0.040030	1.683416	-1.651377

14

E = -306.006158

N	-0.720397	-0.246634	-0.712719
C	-0.603925	1.052041	-0.239663
N	-1.352651	2.148552	-0.268438
C	0.505573	0.398950	0.099095
C	-2.719943	2.129057	-0.768690
H	1.499990	0.411184	-0.321238
C	-0.800800	3.421345	0.174130
C	-1.504934	-1.209370	0.075114
H	-2.953366	1.132015	-1.136562
H	-2.831387	2.837868	-1.595493
H	-3.427989	2.401636	0.022145
H	-0.705721	4.119051	-0.665288
H	0.182846	3.251381	0.610022
H	-1.448674	3.871491	0.932718
H	-2.561584	-1.168517	-0.214084
H	-1.409724	-1.023186	1.153540
H	-1.137054	-2.214214	-0.142061

14H$^+$

E = -306.445191

N	-0.629833	-0.128487	-0.469122
C	-0.636634	1.156013	-0.279478
N	-1.366753	2.223368	-0.267249
C	0.645023	0.497326	-0.004278
C	-2.812899	2.178951	-0.552553
H	0.924504	0.365984	1.040353
C	-0.770361	3.547352	-0.007214
C	-1.391303	-1.339403	-0.167232
H	-3.119271	1.158273	-0.769245
H	-3.025551	2.810878	-1.416200
H	-3.363423	2.550897	0.313096
H	-0.933921	4.190486	-0.873433
H	0.297209	3.439859	0.171974
H	-1.245924	3.990028	0.869205
H	-2.376232	-1.262506	-0.626062
H	-1.489628	-1.469564	0.913832
H	-0.864580	-2.189760	-0.595836
H	1.452957	0.515123	-0.728257

15

E = -345.387466

N	-0.720512	-0.644203	0.662577
C	-0.637769	0.693433	0.165509
N	-1.705579	1.428879	-0.299378
C	0.783494	-0.587980	0.802139
C	0.699939	0.844541	0.310256
C	-1.255868	-1.681583	-0.217623
C	-2.951516	1.349280	0.461481
C	-1.373804	2.764630	-0.772480
H	1.278434	-1.306822	0.140201
H	1.113143	-0.747566	1.833216
H	1.390272	1.671564	0.280286
H	-0.772844	-1.691607	-1.207928
H	-2.327604	-1.531865	-0.364432
H	-1.113958	-2.656796	0.254145
H	-3.112667	0.330322	0.807866
H	-3.787276	1.642828	-0.178302
H	-2.935408	2.009007	1.342802
H	-2.222919	3.172140	-1.325478
H	-0.515424	2.709632	-1.444194
H	-1.130994	3.453935	0.051567

15H$^+$

E = -345.816727

N	-0.539247	-0.606145	0.216113
C	-0.574041	0.713045	0.011124
N	-1.547253	1.582200	-0.138867
C	0.939854	-0.663077	0.313414
C	0.929181	0.868810	0.041520
C	-1.503445	-1.694653	0.340739
C	-2.976566	1.235328	-0.080314
C	-1.245867	2.989591	-0.449226
H	1.392701	-1.281432	-0.462135
H	1.293189	-0.959325	1.301664
H	1.306699	1.493211	0.851222
H	-2.302242	-1.591620	-0.392788
H	-1.923382	-1.742012	1.348370
H	-0.974927	-2.626415	0.140056
H	-3.132741	0.336562	0.509394
H	-3.378259	1.094817	-1.086622
H	-3.508559	2.056123	0.400786
H	-1.703197	3.256652	-1.404190
H	-0.171632	3.143203	-0.516663
H	-1.651901	3.630680	0.335357
H	1.376408	1.171278	-0.905392

16

E = -384.745555

N	-3.686085	0.068053	0.181980
C	-2.822617	1.084840	-0.245585
C	-3.119404	2.284253	-0.784414
N	-1.428883	0.878849	-0.089032
C	-5.072675	0.214330	-0.234911
C	-1.844814	3.061368	-1.011066
C	-0.772956	1.963358	-0.851253
H	-1.724923	3.846029	-0.251616
H	-1.777152	3.551127	-1.987123
H	0.132752	2.302937	-0.343540
H	-0.484729	1.578372	-1.842146
C	-3.566933	-0.295288	1.597466
H	-4.110103	2.703878	-0.869954
C	-0.887752	-0.442682	-0.388498
H	-5.118170	0.383143	-1.311513
H	-5.587063	1.047218	0.272956
H	-5.610394	-0.707958	-0.002609
H	-4.052310	0.449024	2.249111
H	-2.519392	-0.359147	1.885972
H	-4.041135	-1.265656	1.764951
H	-0.959574	-0.685076	-1.462140
H	-1.414547	-1.215721	0.168136
H	0.165959	-0.468531	-0.100131

16H+

E = -385.169376

N	-3.782396	0.319790	0.291468
C	-2.857092	1.150780	-0.170823
C	-3.184210	2.517493	-0.732322
N	-1.543752	0.939565	-0.232959
C	-5.165029	0.778108	0.520800
C	-1.946886	2.845573	-1.585561
C	-0.827487	2.046005	-0.915677
H	-1.731849	3.912367	-1.618056
H	-2.098341	2.500407	-2.609868
H	-0.282619	2.625001	-0.162770
H	-0.104752	1.634017	-1.621591
C	-3.595551	-1.128588	0.489690
H	-4.113297	2.520759	-1.300721
C	-0.732506	-0.061481	0.469166
H	-5.812599	0.470203	-0.304361
H	-5.207419	1.856754	0.640037
H	-5.527469	0.321466	1.442648
H	-3.456712	-1.374350	1.544617
H	-2.757247	-1.495487	-0.094662
H	-4.497619	-1.628265	0.133978
H	-0.462214	-0.890162	-0.188596
H	-1.248937	-0.436120	1.348647
H	0.184592	0.428347	0.798827
H	-3.298417	3.221246	0.100240

Table S2. DFT-calculated enthalpies (*H* in Hartree) and Gibbs energies (*G* in Hartree) at 298.15 K with zero-point energies and thermal corrections included, and entropies (*S* in cal mol^{-1} K^{-1}) for neutral and monoprotonated forms of **1–16**

Compound	Structure	*H*	*G*	*S*
**1**	**E-B$_1$/B$_2$**	-189.299282	-189.332563	70.046
	**Z-B$_1$/B$_2$**	-189.297770	-189.330981	69.898
	**B$_3$**	-189.284285	-189.316528	67.860
	**BH$^+$**	-189.671040	-189.704748	70.944
**2**	**B$_1$/B$_2$**	-266.685961	-266.722212	76.298
	**B$_3$**	-266.667146	-266.702436	74.275
	**BH$^+$**	-267.065312	-267.104729	82.959
**3**	**B$_1$/B$_2$**	-265.510601	-265.546173	74.868
	**B$_3$**	-265.470163	-265.505252	73.852
	**BH$^+$**	-265.877292	-265.913323	75.834
**4**	**E-B$_1$**	-188.036955	-188.067438	64.158
	**Z-B$_1$**	-188.036620	-188.067123	64.198
	**B$_2$**	-188.045257	-188.076709	66.196
	**B$_3$**	-187.986248	-188.018460	67.795
	**BH$^+$**	-188.386444	-188.417812	66.020
**5**	**E-B$_1$**	-227.365807	-227.399003	69.868
	**Z-B$_1$**	-227.364779	-227.397957	69.828
	**B$_2$**	-227.362867	-227.396166	70.085
	**B$_3$**	-227.340439	-227.373661	69.921
	**BH$^+$**	-227.737705	-227.771049	70.177
**6**	**E-B$_1$**	-266.691414	-266.726875	74.633
	**Z-B$_1$**	-266.689878	-266.725342	74.640
	**B$_2$**	-266.693786	-266.729362	74.875
	**B$_3$**	-266.670193	-266.705885	75.121
	**BH$^+$**	-267.072127	-267.107838	75.160

**7**	**E-B$_1$**	-305.987574	-306.025128	79.038
	**Z-B$_1$**	-305.985384	-306.022999	79.168
	**B$_2$**	-305.989211	-306.027050	79.638
	**B$_3$**	-305.972018	-306.011121	82.298
	**BH$^+$**	-306.371883	-306.410066	80.364
**8**	**B$_1$/B$_2$**	-282.750426	-282.785689	74.216
	**B$_3$**	-282.749267	-282.784014	73.130
	**BH$^+$**	-283.133219	-283.169614	76.599
**9**	**B$_1$/B$_2$**	-281.568345	-281.602702	72.310
	**B$_3$**	-281.553521	-281.588570	73.767
	**BH$^+$**	-281.936628	-281.972258	74.990
**10**	**B**	-345.422502	-346.467757	95.249
	**BH$^+$**	-346.818139	-346.864742	98.084
**11**	**B**	-305.919665	-305.960369	85.669
	**BH$^+$**	-306.311255	-306354858	91.772
**12**	**B**	-345.245881	-345.288082	88.821
	**BH$^+$**	-345.650333	-345.696846	97.894
**13**	**B**	-344.051034	-344.094577	91.643
	**BH$^+$**	-344.466947	-344.511239	93221
**14**	**B**	-305.851931	-305.895274	91.223
	**BH$^+$**	-306.275333	-306.319018	91.944
**15**	**B**	-345.201742	-345.245241	91.551
	**BH$^+$**	-345.615867	-345.661208	95.428
**16**	**B**	-384.528998	-384.574135	95.000
	**BH$^+$**	-384.937519	-384.983610	97.008

Table S3. G*n*-calculated enthalpies (*H* in Hartree) and Gibbs energies (*G* in Hartree) at 298.15 K for isomers of **1**, **4**, and **5**

Structure	Method	*H*	*G*
**1-E-B_1/B_2**	**G2**	-188.982183	-189.015356
	**G2MP2**	-188.977570	-189.010743
	**G3B3**	-189.170789	-189.204117
**1-Z-B_1/B_2**	**G2**	-188.980634	-189.013697
	**G2MP2**	-188.976002	-189.009065
	**G3B3**	-189.169286	-189.202388
**1-B_3**	**G2**	-188.965941	-188.998304
	**G2MP2**	-188.961307	-188.993671
	**G3B3**	-189.155579	-189.187967
**1-BH^+**	**G2**	-189.351496	-189.385606
	**G2MP2**	-189.346822	-189.380932
	**G3B3**	-189.540743	-189.574748
**4-E-B_1**	**G2**	-187.722616	-187.753090
	**G2MP2**	-187.718409	-187.748882
	**G3B3**	-187.909555	-187.940155
**4-Z-B_1**	**G2**	-187.722345	-187.752841
	**G2MP2**	-187.718129	-187.748625
	**G3B3**	-187.909430	-187.940057
**4-B_2**	**G2**	-187.731048	-187.762282
	**G2MP2**	-187.726561	-187.757796
	**G3B3**	-187.918311	-187.949632
**4-B_3**	**G2**	-187.670782	-187.702552
	**G2MP2**	-187.666634	-187.698404
	**G3B3**	-187.858345	-187.890521
**4-BH^+**	**G2**	-188.069243	-188.100786
	**G2MP2**	-188.065012	-188.096556
	**G3B3**	-188.256902	-188.288371
**5-E-B_1**	**G2**	-226.981116	-227.014265
	**G2MP2**	-226.975946	-227.009095
**5-Z-B_1**	**G2**	-226.980093	-227.013266
	**G2MP2**	-226.974905	-227.008078
**5-B_2**	**G2**	-226.979729	-227.013083
	**G2MP2**	-226.974486	-227.007840
**5-B_3**	**G2**	-226.956319	-226.989586
	**G2MP2**	-226.951197	-226.984465
**5-BH^+**	**G2**	-227.351134	-227.384619
	**G2MP2**	-227.345966	-227.379451

Table S4. DFT-calculated relative enthalpies, entropy terms, Gibbs energies (ΔH_{298}, $T\Delta S_{298}$, and ΔG_{298} in kJ mol^{-1}, respectively), tautomeric equilibrium constants (K), and percentage contents (%) for neutral tautomeric bases **1-9**

Structure	Method	ΔH_{298}	$T\Delta S_{298}$	ΔG_{298}	K	%
**1-*E*-B$_1$/B$_2$**	**DFT**	0.00	0.00	0.00	1.000	84.230
**1-*Z*-B$_1$/B$_2$**	**DFT**	3.96	-0.18	4.15	0.187	15.770
**1-B$_3$**	**DFT**	39.37	-2.73	42.10	4.2 10^{-8}	3.5 10^{-6}
**2-B$_1$/B$_2$**	**DFT**	0.00	0.00	0.00	1.000	100
**2-B$_3$**	**DFT**	49.40	-2.52	51.92	8.0 10^{-10}	8.0 10^{-8}
**3-B$_1$/B$_2$**	**DFT**	0.00	0.00	0.00	1.000	100
**3-B$_3$**	**DFT**	106.17	-1.27	107.44	1.5 10^{-19}	1.5 10^{-17}
**4-*E*-B$_1$**	**DFT**	21.80	-2.54	24.34	5.4 10^{-5}	0.005
**4-*Z*-B$_1$**	**DFT**	22.68	-2.49	25.17	3.9 10^{-5}	0.004
**4-B$_2$**	**DFT**	0.00	0.00	0.00	1.000	99.991
**4-B$_3$**	**DFT**	154.93	1.99	152.93	1.6 10^{-27}	1.6 10^{-25}
**5-*E*-B$_1$**	**DFT**	0.00	0.00	0.00	1.000	72.473
**5-*Z*-B$_1$**	**DFT**	2.70	-0.05	2.75	0.330	23.935
**5-B$_2$**	**DFT**	7.71	0.27	7.45	0.049$_5$	3.592
**5-B$_3$**	**DFT**	66.60	0.07	66.54	2.2 10^{-12}	1.6 10^{-10}
**6-*E*-B$_1$**	**DFT**	6.23	-0.30	6.53	0.072	6.611
**6-*Z*-B$_1$**	**DFT**	10.26	-0.29	10.55	0.014	1.304
**6-B$_2$**	**DFT**	0.00	0.00	0.00	1.000	92.085
**6-B$_3$**	**DFT**	61.94	0.31	61.64	1.6 10^{-11}	1.2 10^{-9}
**7-*E*-B$_1$**	**DFT**	4.30	-0.75	5.05	0.131	11.414
**7-*Z*-B$_1$**	**DFT**	10.05	-0.59	10.64	0.014	1.197
**7-B$_2$**	**DFT**	0.00	0.00	0.00	1.000	87.389
**7-B$_3$**	**DFT**	45.14	3.32	41.82	4.7 10^{-8}	3.8 10^{-6}
**8-B$_1$/B$_2$**	**DFT**	0.00	0.00	0.00	1.000	85.495
**8-B$_3$**	**DFT**	3.04	-1.35	4.40	0.170	14.505
**9-B$_1$/B$_2$**	**DFT**	0.00	0.00	0.00	1.000	100
**9-B$_3$**	**DFT**	38.92	1.82	37.10	3.2 10^{-7}	3.2 10^{-5}
